# A Spring Forward for Hominin Evolution in East Africa

**DOI:** 10.1371/journal.pone.0107358

**Published:** 2014-09-10

**Authors:** Mark O. Cuthbert, Gail M. Ashley

**Affiliations:** 1 Connected Waters Initiative Research Centre, UNSW Australia, Sydney, NSW, Australia; 2 School of Geography, Earth and Environmental Sciences, University of Birmingham, Birmingham, United Kingdom; 3 Department of Earth and Planetary Sciences, Rutgers University, Piscataway, New Jersey, United States of America; University College London (UCL), Canada

## Abstract

Groundwater is essential to modern human survival during drought periods. There is also growing geological evidence of springs associated with stone tools and hominin fossils in the East African Rift System (EARS) during a critical period for hominin evolution (from 1.8 Ma). However it is not known how vulnerable these springs may have been to climate variability and whether groundwater availability may have played a part in human evolution. Recent interdisciplinary research at Olduvai Gorge, Tanzania, has documented climate fluctuations attributable to astronomic forcing and the presence of paleosprings directly associated with archaeological sites. Using palaeogeological reconstruction and groundwater modelling of the Olduvai Gorge paleo-catchment, we show how spring discharge was likely linked to East African climate variability of annual to Milankovitch cycle timescales. Under decadal to centennial timescales, spring flow would have been relatively invariant providing good water resource resilience through long droughts. For multi-millennial periods, modelled spring flows lag groundwater recharge by 100 s to 1000 years. The lag creates long buffer periods allowing hominins to adapt to new habitats as potable surface water from rivers or lakes became increasingly scarce. Localised groundwater systems are likely to have been widespread within the EARS providing refugia and intense competition during dry periods, thus being an important factor in natural selection and evolution, as well as a vital resource during hominin dispersal within and out of Africa.

## Introduction

Hominin fossil discoveries in the last few decades have shown that humans evolved in Africa and then migrated in waves to other parts of the world, starting as early as 1.85 Ma [1]. Both marine and terrestrial records in the region point to a general increase in aridity during the Plio-Pleistocene [2]–[4] primarily due to progressive rifting in East Africa and associated tectonic uplift [5]. The climate during the last 6–7 million years when hominins evolved to modern humans was characterized by high variability [6], [7]. In the tropics of Africa it was dominated by wet and dry cycles driven by orbital forcing and, in particular, the effect of precession on monsoon strength [8]–[11]. The climate is also likely to have varied on the millennial timescale due to tropical expressions of Dansgaard-Oeschger cycles [12], as well as on a decadal timescale governed by variations in El Nino Southern Oscillation (ENSO) and Indian Ocean Dipole (IOD) variations [13], [14]. A major unknown connected with human evolution in this climatically turbulent environment [6] is the availability of resources, particularly freshwater. Recent research has suggested the importance of freshwater availability from some lakes for hominin survival and dispersal in the EARS [6], [11], [15], [16]. Finlayson [17] even suggests that “the need for swift and efficient movement between ever shrinking sources of water” was *the* trigger for human evolution. However, many of the EARS lakes are saline and were likely so in the past, thus their potability, during periods of hominin radiations in the last 5 Ma, is speculative [11], [18]. Furthermore, streams which originate in arid and semi-arid areas are ephemeral in nature; perennial flows are only possible in large catchments where humid source areas upstream contribute consistent inflows via runoff and groundwater baseflow [19]. During periods of increased aridity, sources of riverine or potable lake water would therefore have been scarce in many parts of the EARS.

Groundwater is protected from evaporation and thus potentially provides a key alternative potable resource for sustaining life through drought periods in areas with variable rainfall [20]. Hence, springs and groundwater-fed habitats could have played a decisive role in the survival and dispersal of hominins during these times of known climate variability [6], [15] when potable surface water was limited. The significance of groundwater in this context has, nevertheless, received little attention, although geological evidence for active freshwater springs has recently been linked to archaeological hominin remains at Olduvai Gorge [21], [22] and the first fossil chimpanzee [23] elsewhere in the EARS.

**Figure 1:**
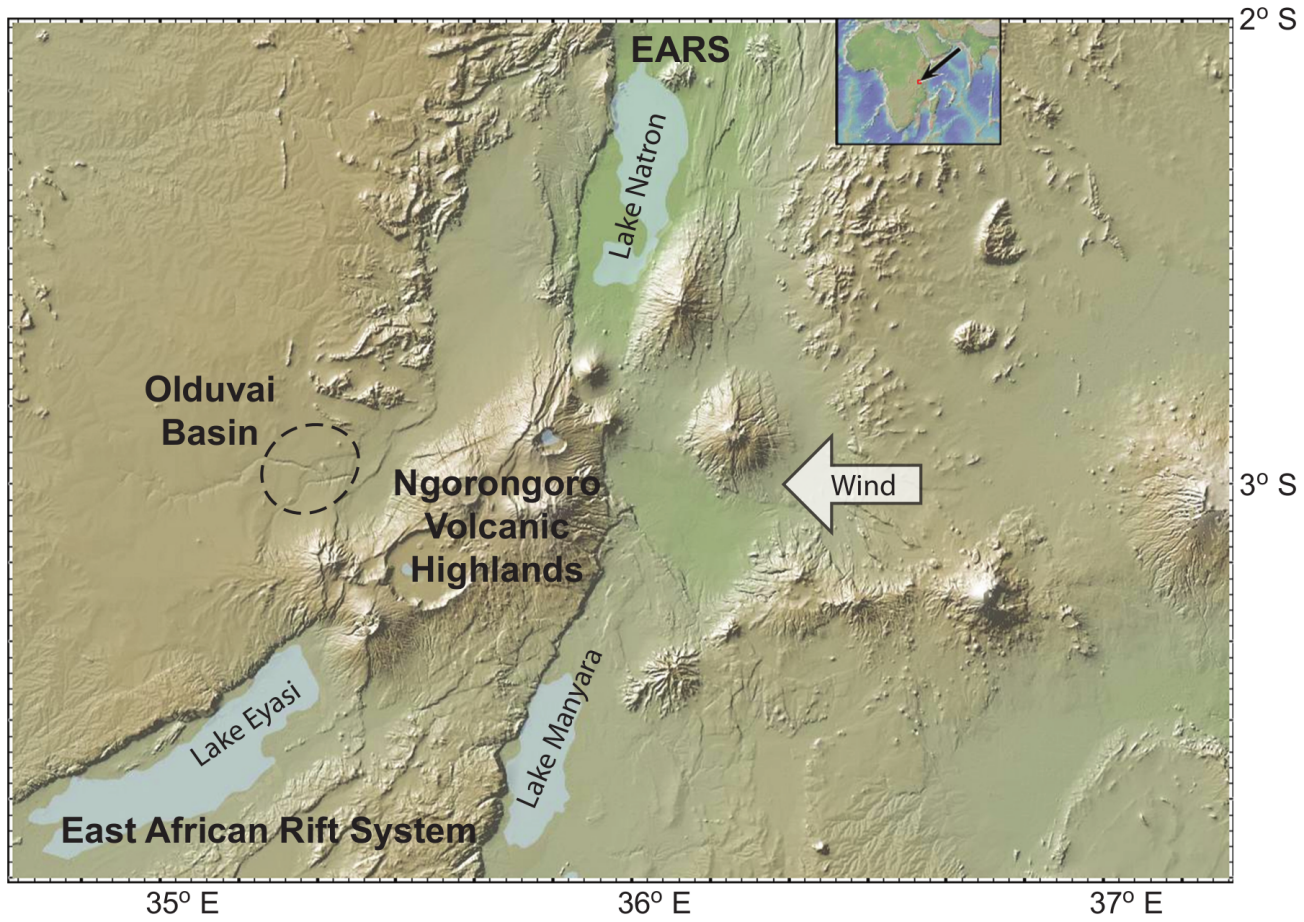
Location map. Insert shows with arrow the location of study area in eastern Africa. Map of the Northern Tanzanian Divergence Zone depicts the East African Rift System (EARS), containing Lake Natron (north), diverging around the Ngorongoro Volcanic Highland massif and splitting into two separate rift valleys (Lake Eyasi on west) and Lake Manyara (on east). Prevailing wind is from the east. Olduvai basin lies to the west of and in the rain shadow of Ngorongoro. (Map made by Sara Mana, http://www.geomapapp.org).

Despite the geological evidence for the presence of springs, it is presently unclear how such spring discharges may have varied with climate. Our objectives are therefore to provide estimates for the temporal variability of spring flow at Olduvai Gorge, at around 1.8 Ma BP under different climate variability scenarios and to consider the wider implications of this new information in the context of current hypotheses about human evolution in the East African Rift System (EARS) (Figure 1, http://www.geomapapp.org).

## Physical Setting

### Geology

**Figure 2:**
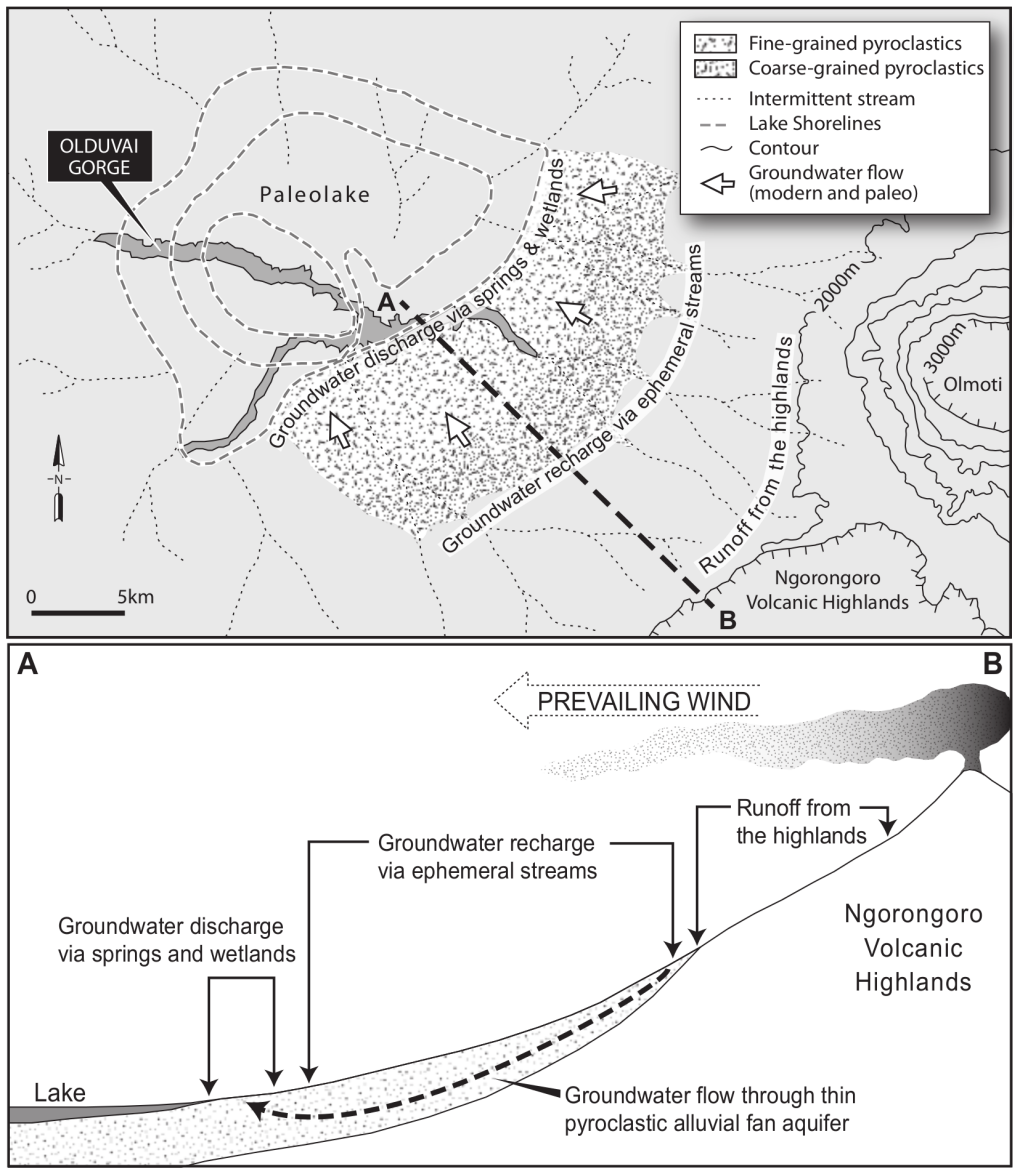
Paleoenvironmental reconstruction of Olduvai and schematic hydrogeological conceptual model (modified from [28]).

The Olduvai sedimentary basin was formed ∼2.2 Ma years ago on the western margin of the EARS, at 3°S, in response to the growth of a large volcanic complex (Ngorongoro) (Figure 1). The region is in the Northern Tanzanian Divergent Zone, a prominent bifurcation in the main rift valley. Ngorongoro Volcanic Highland is a ∼4,000 km^2^ massif comprised of 8–10 eruptive centers of alkali magma compositions [24], [25]. The paleo basin is an estimated 3,500 km^2^ in area and bordered on the south and east by the volcanic highlands and on the west and north by metamorphic terrain. The preserved sedimentary record in the basin center is 30 km in diameter and composed of 100 meters of interbedded pyroclastics (tuffs), volcaniclastic sediments and minor limestones. A playa lake occupied the center of the basin during the first million years, seasonal rivers drained the periphery and an alluvial fan comprised of fine- and coarse-grained pyroclastics fringed the basin margin on east and south (Figure 2).

Olduvai Basin is ideal for studying paleohydrology. The tuffs are precisely dated using ^40^Ar/^39^Ar single crystal method [26] and can be traced through the basin using tephrocorrelation [27]. The variation in stratigraphic sequences was documented throughout the basin [25], [28]. Field mapping identified the lithologic record of Milankovitch (precession) driven lake cycles [8], [29] and the location of groundwater-fed environments. The paleoenvironmental reconstruction was based on sedimentary facies and structures [30], [31], clay mineralogy [32], [33], stable isotopes [21], [34], [35], and fossilized plant remains [31], [36]–[38]. The association of spring and wetland deposits is documented in deposits 2 to 1 Ma in age [39]–[42].

### Hydrology

**Figure 3:**
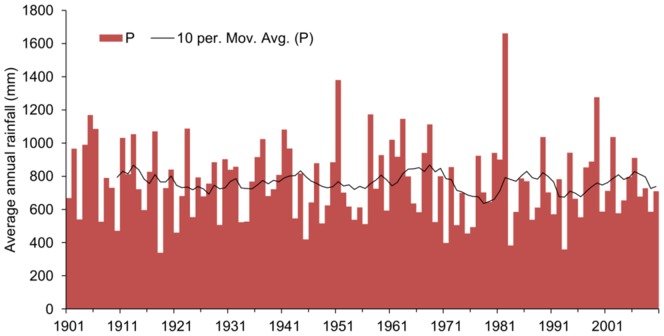
Gridded annual precipitation data (P) from the Global Precipitation Climatology Centre (GPCC) for grid square 2.75 deg S, 35.25 deg E in which Olduvai Gorge is situated. A 10 year moving average is also plotted. Data was accessed on 26/4/13from: http://iridl.ldeo.columbia.edu/SOURCES/.WCRP/.GCOS/.GPCC/.FDP/.version6/.

The shallow Olduvai basin contained a saline-alkaline lake flanked to the east and south by freshwater wetlands [21]. Two rainy seasons currently provide around 550 mm/a of precipitation to the Olduvai lowland and around twice that in the highlands due to the elevation difference and rain-shadow effect [36]. This difference was also likely present in the past since the topographical contrast was similar. Annual rainfall data show substantial interannual and decadal variability for the 20^th^ century (Figure 3). Around 1.8 Ma, wet-dry cycles driven by precession (21 ka) and modulated by eccentricity (100 ka) (orbital monsoon hypothesis [7]) were superimposed on a longer term drying trend [43]. There were 5 wet and 5 ½ dry cycles between 1.85–1.74 Ma [43] during which the seasonal variability in insolation, and thus also temperature, potential evapotranspiration and monsoon strength [8], would have fluctuated. Annual rainfall is thought to have varied from 250 to 700 mm/a during arid and wetter intervals respectively [44]. Shorter term (years to decades) variations also occurred [35] with centennial to millennial variability also likely. The strengthening of the Walker circulation around this time [13] is also likely to have led to more extreme climate variability on the annual to decadal timescale. This may have been particularly significant for providing an intensification of rainfall and therefore increased frequency of runoff-recharge events [14], [45].

Our conceptual model of the hydrology of paleo-Olduvai is as follows. Higher rainfall and steep slopes on the flanks of the Ngorongoro Volcanic Highland caused significant runoff feeding the upper parts of a pyroclastic alluvial fan at the mountain front (Figures 1 and 2). This is likely to have occurred under intense rainfall conditions even when potential evapotranspiration in the region was high (likely >2000 mm/a [14], [22]). Ephemeral stream flow then led to ‘indirect’ groundwater recharge through the base of the streams into the fan deposits [43], [46]. ‘Diffuse’ groundwater recharge across the wider region was unlikely given the predominant semi-arid climate conditions unless there were occasional very prolonged and intense rainfall events. Such events may have enabled soil moisture deficits to be overcome locally and/or triggered preferential flow pathways through the soils to enable water to escape the zone of evapotranspiration [14], [47]. How far down the fan the ephemeral streams flowed during flow events would have been a complex interaction between antecedent moisture conditions, streambed permeability and the magnitude of the flow event [46]. However, initial groundwater mounding underneath the stream channels would have spread transversely into the fan deposits, at the same time as flowing longitudinally downslope to the lower parts of the fan. Flow-focussing via faults and the juxtaposition of lower permeability deposits on the fan margin led to local spring discharges and significant tufa deposition [21].

## Groundwater Model

### Model Formulation

The objective of the modelling is to provide the likely range of timescales for groundwater discharge recession for the paleo-Olduvai groundwater system. The models have been designed to contain, conceptually, the most important features of the flow system while being kept mathematically simple enough to enable analytical solutions to the problems to be found. This enables the large range of parameter uncertainty to be analysed without excessive computational effort while also making it possible to test a variety of different boundary conditions to cover the necessary groundwater recharge input scenarios.

**Figure 4:**
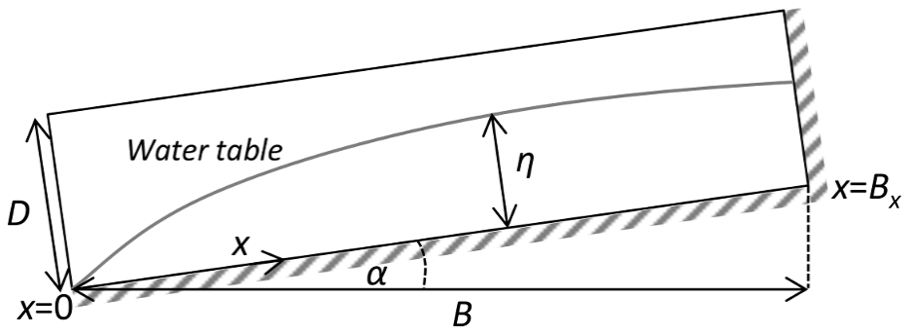
Simplified conceptual groundwater flow model for a sloping aquifer.



Numerical models were developed based on analytical solutions to the following 1–D linearised Boussinesq equation for groundwater flow through a homogeneous and isotropic sloping aquifer [48] sketched conceptually in Figure 4:

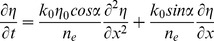
(1)where *t* is time [T], *x* is distance along the aquifer base [L], *η* is hydraulic head [L], *k_0_* is aquifer hydraulic conductivity, *n_e_* is aquifer specific yield [−], *α* is the slope of the aquifer [−], *η_0_* is the average water table height equal to 0.3*D* where *D* is the maximum saturated thickness of the aquifer [L].

It is assumed that the lateral spreading occurring away from the ephemeral streambeds during recharge events occurs at timescales orders of magnitude faster than the longitudinal drainage of the fan. This is reasonable since the groundwater response time is controlled by *L^2^n_e_/T* [49] and the lateral spreading occurs over a length scale (*L*) much smaller than the length of the fan longitudinally. Thus, groundwater recharge (*I* [LT^−1^]) is assumed to be evenly distributed across the domain between *x* = 0 at the spring discharge point which is represented by a constant head boundary, and a groundwater divide represented by a no flow boundary at *x* = *B_x_* (Figure 4). The total recharge input is thus equal to *IB* where *B* is the horizontal distance between the constant head and no flow boundaries. Two boundary value problem scenarios are solved for the time variant groundwater discharge (*q* [L^3^T^−1^]) at *x = *0 as follows.

#### Scenario 1: Sudden cessation of recharge

The first case considered is the sudden cessation of recharge after a wet period when the aquifer is at a steady state. An existing solution to equation (1) for the stated boundary conditions which proves very useful for the Olduvai case is as follows [48]:
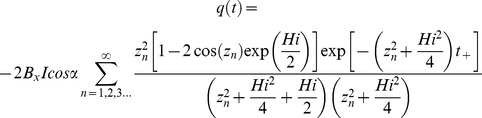
(2)with *z*_n_ being the n^th^ root of tan(*z*) = 2*z*/*Hi* and:



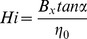
(3)

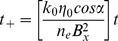
(4)


#### Scenario 2: Periodic variation of recharge

No solution to equation (1) for the stated boundary conditions exists in the literature to calculate the outflow for the case of a periodic variation in recharge in a sloping aquifer. However, using Brutsaert’s [48] solution for a unit response to a step change in recharge as follows:
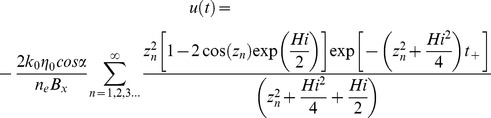
(5)and then applying a convolution integral for a periodic variation around some average recharge (*I_av_*), with:
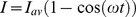
(6)leads to the following equation for the outflow at *x* = 0:



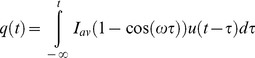
(7)The solution to this integral is:
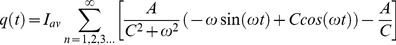
(8)with
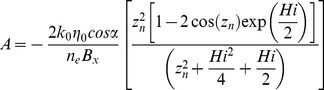
(9)and



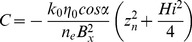
(10)Model outputs have been plotted as amplitude ratios (AR) and phase shifts (PS) normalised to the recharge input (i.e. using *q*/(*I_av_.B*)) and the period of oscillation (*P* = 2*π*/*w*) respectively as follows:

AR = (amplitude of *q*)/(amplitude of *I_av_.B*)

PS = time lag between maxima (or minima) in *q*, and maxima (or minima) in *I_av_.B*/P

The AR is thus a measure of how much the outflow varies in comparison with the input signal; the PS is how much the output signal lags the input signal as a proportion of the input period. Smaller ARs mean the groundwater outflow is highly damped in comparison to the input recharge signal and this is normally accompanied by larger PS.

**Figure 5:**
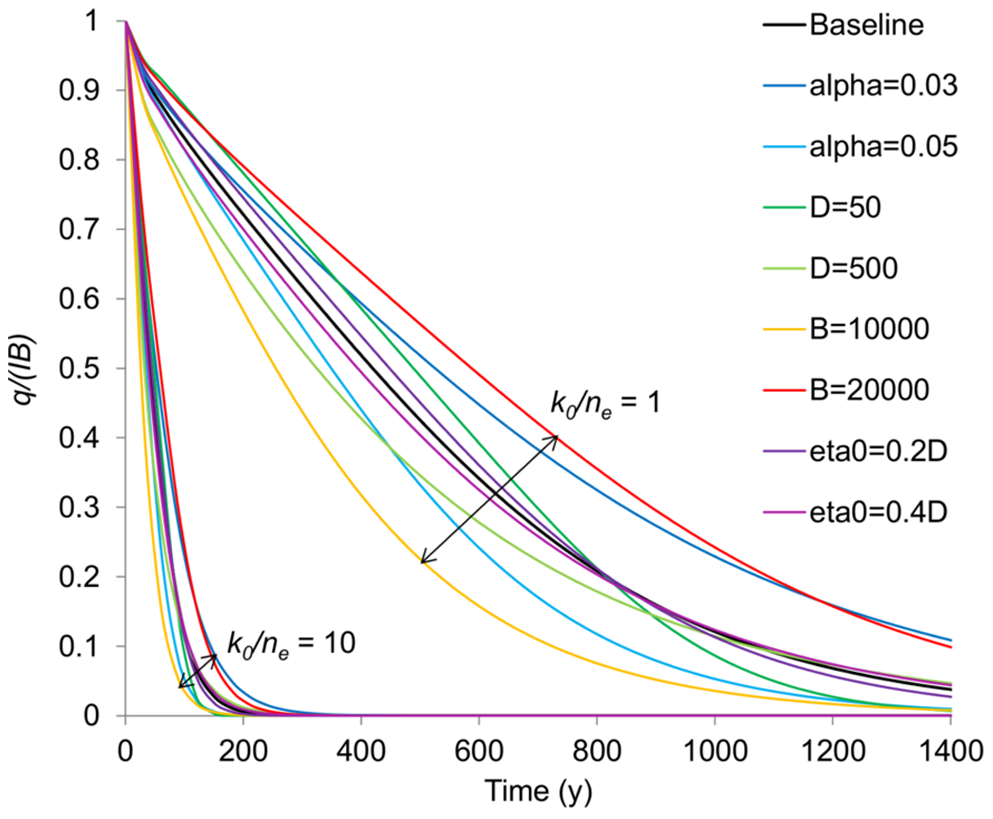
Geometric parameter sensitivity of spring flow recession using **equation (2)**. Variation in recession rates is predominantly controlled by variations in the ratio of hydraulic conductivity to specific yield (*k_0_/n_e_*) and relatively insensitive to variations in the geometry, i.e. the spread *around* the baseline recession for the two *k_0_/n_e_* end members (*k_0_/n_e_* = 1 and *k_0_/n_e_* = 10) is much smaller than variation *between* the end members.

**Figure 6:**
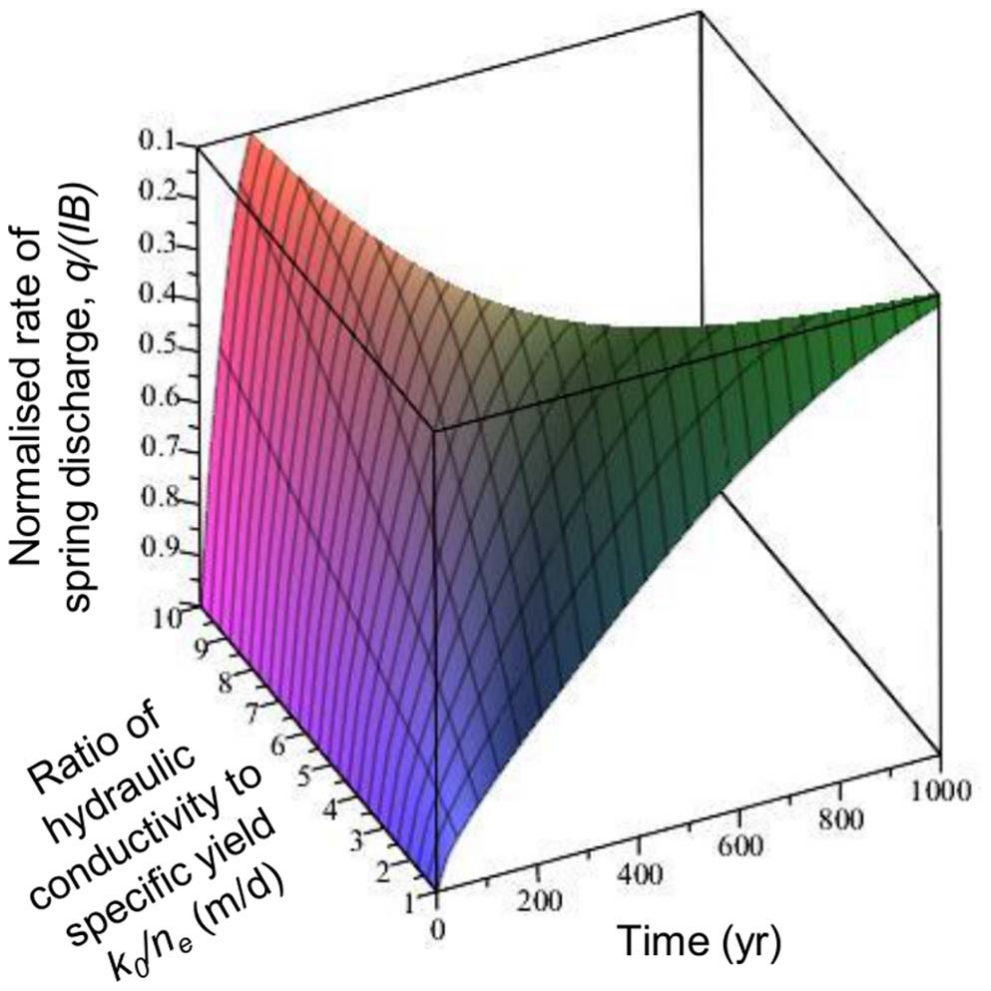
Rate of groundwater discharge (*q,* modelled using Eq. 2) at Olduvai springs normalised to the rate of groundwater recharge (*IB*) for the case of a sudden cessation of groundwater recharge after a period of steady state. Baseline geometry parameters have been used and hydraulic properties (*k_0_/n_e_*) were varied across the likely range. The vertical axis, *q/(IB)*, is clipped at 0.1 to illustrate the range of timescales by which the discharge recedes to 10% of its original value.

**Figure 7:**
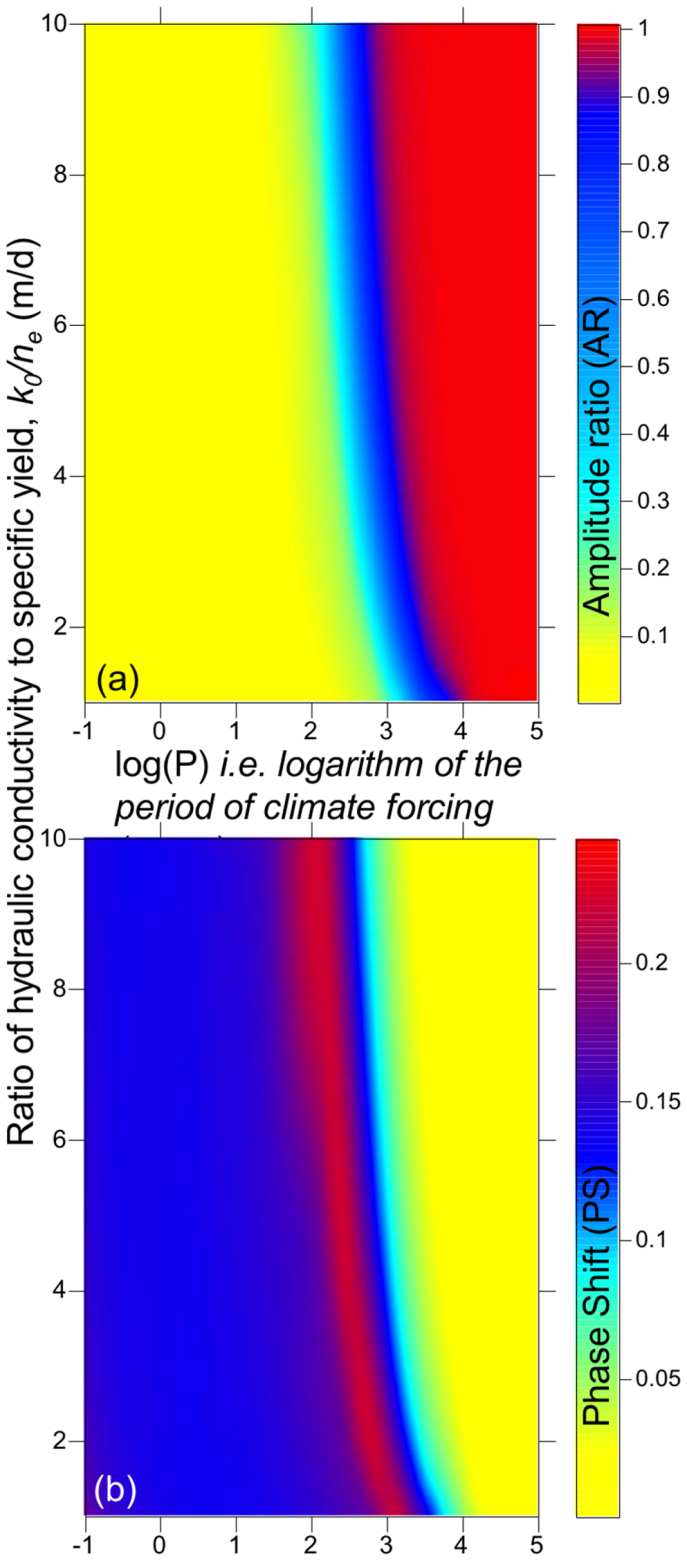
Modelled (a) amplitude ratio (AR) and (b) phase shift (PS) of the input groundwater recharge forcing relative to the spring discharge output on periods from 0.1 year to 100 000 years across a range of likely aquifer hydraulic properties (using Eq. 8–10). The transition to lower amplitude ratios and higher phase lags for periods lower than 100 to 1000 years implies greater buffering of the climate signal and increased potential resilience of the spring discharge to climate variability on these timescales.

The analytical solution, Eq. 2–4, used for the first ‘sudden cessation of recharge’ scenario is a well-known published result [48]. Since the analytical solution for the periodic case was derived for this paper (Eq. 8–10) it was therefore tested against a published solution for the end member where the aquifer is not sloping [50], [51] and agreed perfectly. Although it is unlikely that recharge varied exactly as a step function (Scenario 1) or exactly sinusoidally (Scenario 2), the two solutions have been chosen to provide plausible and contrasting end members of the possible recharge variability under likely variations in climate. The software Maple (v.17) was used for the model calculations; the model files and the model data used to produce Figures 5 to 7 are included as Dataset S1.

### Model Parameterisation

The geometry of the alluvial fan and thus the length scale of the groundwater flow system (*B*) is relatively well constrained from the paleo-reconstruction and is likely to have been in the range 15 km +/−5 km. The slope angle (α) is likely to have been in the range 0.04+/−0.01.

While there are relatively few detailed studies on the hydraulic characterisation of pyroclastic materials, a literature review (Table 1) suggested [52]–[57] that a range of *k_0_* of 0.05 to 5 m/d is reasonable (for length scales of 10 s of km) in combination with likely saturated thicknesses of <200 m for the fan materials to keep *T*<1000 m^2^/d consistent with the literature values. Specific yield of porous materials is normally in the range 0.05 to 0.5. However, covariation in porous materials of *k_0_* and *n_e_* leads to a likely range of *k_0_*/*n_e_* between minimum 0.05/0.05 = 1 and maximum 5/0.5 = 10 which was used for all the models.

**Table 1:** Range of literature values for volcanically derived aquifers used to parameterise the groundwater models.

Reference	Range of hydraulic properties: hydraulic conductivity (*k_0_*, m/d), transmissivity (*T*, m^2^/d) and specific yield (*n_e_*, -)
Gleeson *et al.* [54]	*k_0_ ≈* 0.5 for mid-range ‘regional’ volcanic values
Yihdego & Becht [57]	*k_0_ ≈* 0.1 to 0.4 for reworked volcanics near Lake Naivasha EARS
Belcher *et al.* [53]	*k_0_ ≈* 0.04 to 4 for Death Valley tuffs
Ayenew *et al.* [52]	*T ≈* 27 to 135 in Ethiopian EARS
Greco *et al.* [55]	*k_0_ ≈* 7 for Italian shallow pyroclastic deposits
Moghaddam & Najib [56]	*k_0_ ≈* 1.05 to 3.5, *T ≈* 242 to 858 for Iranian tuff

A sensitivity analysis for the geometric parameters used in the models (*α*, *D*, *B* and *η_0_*) was carried out by altering each parameter across its likely range away from a baseline parameter set, and observing the change in the recession timescale using Eq. 2–4. The baseline parameters are as follows: *B* = 15 km, α = 0.04, *η_0_* = 0.3*D*, *D* = 200 m with sensitivity ranges as defined in Figure 5. Of the geometric parameters, the rate of recession is most sensitive to the length of the flow system, *B*. However, it is clear that the sensitivity to the geometric parameters is insignificant in comparison to the sensitivity to the hydraulic diffusivity (ratio of *k_0_*/*n_e_*) (Figure 5). The results we present and discuss in the paper, despite having a fixed geometry, are thus illustrative of the likely range of hydrodynamic characteristics.

## Results and Discussion

### Limits of Spring Longevity at Olduvai

Even in the present day it is often impossible to estimate groundwater recharge rates to within an order of magnitude in semi-arid areas solely on the basis of known climate variables [58]. Based on the record of extensive tufa deposition in spring and associated wetland environments at Olduvai between 1.84 to 1.36 Ma [21], [30] we can be sure that freshwater would have been flowing actively and thus available for direct consumption even, to some extent, during the driest periods of the precessional cycles. However, temporally, the geological record is discontinuous and it is not possible to deduce how persistent the spring flows may have been on the basis of the geology alone. Whereas it is not possible to be precise about the absolute rates of recharge and discharge, the groundwater models we have developed here, based on our paleoenvironmental reconstruction and hydrological conceptual model, are able to realistically estimate the plausible range of variation in flows as well as their persistence during dry periods. As would be expected intuitively, as aquifer hydraulic diffusivity increases, modelled spring flows become more responsive to the climate forcing and have characteristically shorter recessions (periods of declining flow). For the case of a sudden reduction of groundwater recharge during an extended drought period, across the likely range of hydraulic properties (*k_0_*/*n_e_*), spring discharge recedes to almost zero flow after approximately 150 to >1000 years (Figure 6). This suggests that the Olduvai springs and wetland would likely have continued to be supported by groundwater flow for a few 100 to approx. 1000 years even with no recharge.

For the case of a periodic variation in the groundwater recharge forcing, as the period increases the amplitude ratio (AR) increases indicating decreased damping, irrespective of the hydraulic diffusivity (Figure 7). The phase shift (PS) shows a more complex relationship with period which is more strongly dependent on the diffusivity. For 20 ka Milankovitch recharge cycles, the spring discharge lags recharge by a few hundred years and is almost identical in amplitude (Figure 7). Once the period is shorter than around 2 ka, discharge maxima and minima lag those of the forcing recharge signal by up to 400 years for the lower hydraulic diffusivity end member and are damped by as much as 50% of the magnitude of the recharge variation. This means that groundwater would have been available during the driest periods of millennial climate cycles, only diminishing significantly on the rising limb when potable surface water sources may have become more plentiful. For recharge oscillations on the order of 100 years or less there is almost complete damping of the input signal (Figure 7) meaning that groundwater discharge would have varied by less than 20% during droughts over these timescales despite large changes in groundwater recharge.

Considering the superposition of climate variations occurring at different frequencies, these results indicate that, for multi-millennial and greater period variations, the wetland springs would only have provided a refugium if sufficient groundwater recharge still occurred at centennial or higher frequencies during the driest parts of longer term climate cycles. At Olduvai, this is likely to have been the case since there is stratigraphic evidence of some tufa deposition during dry parts of the 21 ka climate oscillation [22]. Sufficient, albeit likely sporadic, groundwater recharge must have been occurring despite the arid conditions. The recharge was made possible due to the focussing effect of runoff on the highland flank which caused indirect recharge through the base of ephemeral stream channels. This phenomenon is well known to occur in similar modern day arid settings [58]–[61].

Therefore, in combination, the geology and the modelling suggest that the groundwater system at Olduvai would have provided a freshwater resource throughout the precessional cycle even during long droughts occurring on decadal to multi-centennial timescales.

### Wider availability of groundwater within the EARS

While Olduvai Gorge was the first archaeological locality at which geological evidence of groundwater discharge was recognised, other localities within the EARS have recently been identified [62], [63]. Many semi-arid EARS settings in the present day have active groundwater systems [64], [65] providing seeps and springs supporting vegetation, watering holes and lakes, and it is likely that they also would have done so in the past in similar settings and provided essential freshwater resources during dry periods to hominins and other animals. Many of the EARS lakes during the critical hominin radiation periods appear to have been saline [11], [18] and we propose that groundwater springs may have provided important ‘landscapes for evolution’ [66] in the wider context of the EARS in addition to any surface water sources that may have also been potable. It has been suggested that that it took about 2 ka for the large EARS lakes to completely dry up [16]. However, during such dry periods it is possible that, while runoff in such catchments was not hydrologically effective enough at the basin scale to keep a lake wet, localised groundwater recharge could still feed spring systems and provide critical freshwater resources. The modelling results presented here show, for the first time, the likely timescale for the springs at Olduvai to remain active with no rainfall was in the range 100 s–1000 years, and geological evidence points to the fact that the springs were active during the driest times of the precessional cycle. Other sites in similar settings in the EARS may have been more or less ‘drought proof’ in comparison to Olduvai. For example our model sensitivity analysis shows that increased length scales, decreased diffusivity, increased thickness or more gentle slopes in a groundwater system leads to increased buffering of the climate signal (Figure 5). Different catchment geometries and runoff characteristics would have also led to variations in recharge thresholds with varying aridity across the EARS. In catchments without steep slopes or increased rainfall at higher elevations, indirect recharge may not have occurred frequently, and recharge may only have been possible during periods of intense rainfall for example driven by particularly strong El Nino events [14].

### Implications for Hominin Evolution and Dispersal

There is a broad coincidence between aridification of Africa (and the associated expansion of open woodlands and grasslands [2]) over the last 7 Ma, and the development of bipedalism. Increasing climate variability at about 2.5 Ma is associated with the first record of genus Homo, the first appearance of stone tools (evidence of technological capabilities) [67], the increase in cranial capacity [68], and eventually the migration of hominins out of Africa. The development of complex cognitive processes (e.g. language) and art [69] cannot be tied directly to climate. However, there have been several attempts to explain the role of past climate change on hominin evolution which focus on the importance of climate instability. For example, Potts’ “variability selection” theory proposes that dramatic climatic shifts favoured animals that were truly generalists and could adapt to a wide range of environmental conditions [70]. More recently, in a variation on this theme, the “pulsed climate variability hypothesis” [71] argues for extreme wet-dry cycles, in particular the precession-driven appearance and disappearance of deep EARS lakes, driving hominin evolution. While around 2 Ma most EARS lakes dried up or became saline during arid precessional phases, a notable exception is that of Lake Turkana, due its extensive catchment in the Ethiopian Highlands, which may have provided a continuous “aridity-refugium” during the whole precessional cycle [72].

The major contribution we propose here to this debate is in showing how groundwater refugia could also have persisted during the driest parts of the precessional cycle. We hypothesise that as surface water sources became more scarce during a given precessional cycle, the only species to survive may have been those with adaptations for sufficient mobility to discover a new and more persistent groundwater source, or those already settled within home range of such a resource. Such groundwater refugia may have been sites for intense competition between hominin and other animal species and hence selective pressure favouring those who could maintain access to water, something for which there is no substitute. Furthermore we speculate that, during wetter periods, springs may have formed ways of ‘bridging’ longitudinal dispersal of hominins between larger freshwater bodies or rivers providing a critical resource during hominin migration within and out of Africa.

Thus we consider that, while the argument for the persistence of springs during arid periods is robust, further exploration is needed to test hypotheses as to how groundwater flow systems produced by the EARS played a significant role in the evolution and dispersal of humans in the region.

## Supporting Information

Dataset S1 Model files and data used to generate Figures 5 to 7.
(ZIP)
